# Comparative transcriptomics analysis pipeline for the meta-analysis of phylogenetically divergent datasets (CoRMAP)

**DOI:** 10.1186/s12859-022-04972-9

**Published:** 2022-10-07

**Authors:** Yiru Sheng, R. Ayesha Ali, Andreas Heyland

**Affiliations:** 1 Department of Mathematics and Statistics, University of Guelph, Guelph, N1G 2W1 Canada; 2 Integrative Biology, University of Guelph, Guelph, N1C-1A8 Canada

**Keywords:** Gene expression, Brain, RNA-Seq, Diversity

## Abstract

**Background:**

Transcriptional regulation is a fundamental mechanism underlying biological functions. In recent years, a broad array of RNA-Seq tools have been used to measure transcription levels in biological experiments, in whole organisms, tissues, and at the single cell level. Collectively, this is a vast comparative dataset on transcriptional processes across organisms. Yet, due to technical differences between the studies (sequencing, experimental design, and analysis) extracting usable comparative information and conducting meta-analyses remains challenging.

**Results:**

We introduce Comparative RNA-Seq Metadata Analysis Pipeline (CoRMAP), a meta-analysis tool to retrieve comparative gene expression data from any RNA-Seq dataset using de novo assembly, standardized gene expression tools and the implementation of OrthoMCL, a gene orthology search algorithm. It employs the use of orthogroup assignments to ensure the accurate comparison of gene expression levels between experiments and species. Here we demonstrate the use of CoRMAP on two mouse brain transcriptomes with similar scope, that were collected several years from each other using different sequencing technologies and analysis methods. We also compare the performance of CoRMAP with a functional mapping tool, previously published.

**Conclusion:**

CoRMAP provides a framework for the meta-analysis of RNA-Seq data from divergent taxonomic groups. This method facilitates the retrieval and comparison of gene expression levels from published data sets using standardized assembly and analysis. CoRMAP does not rely on reference genomes and consequently facilitates direct comparison between diverse studies on a range of organisms.

**Supplementary Information:**

The online version contains supplementary material available at 10.1186/s12859-022-04972-9.

## Background

Transcriptional regulation is vital for all living organisms to orchestrate biological processes and the quantitative analysis of transcriptional changes in space and time has provided fundamental insights into organismal functions. For example, gene expression and its regulation have long been implicated as major players in the formation of long-term memory [1, 2]. Numerous studies have not only supported this hypothesis but have also provided evidence for the conservation of specific genes and pathways in this process. However, most information on the involvement of transcriptional changes in learning and memory originates from a small number of model organisms. The identification of lineage specific genes requires a robust comparative framework as well as sufficient information on gene expression levels across multiple species [3].

High throughput RNA sequencing technologies (RNA-Seq) are a precise, highly efficient, and cost-effective tool to conduct and analyze whole genome transcriptomes, allowing for the detection of the transcriptomic response of organisms under various environmental or disease conditions [4–8]. Furthermore, a diverse array of tools has been developed for quality control, quality improvement, sequence assembly, quantification of gene expression, differential expression analysis, gene annotation, as well as the analysis of pathways, gene regulation mechanisms and functional groups [9–12]. Still, various factors limit the meta-analysis of RNA-seq studies. Among these are variations in experimental design, sequencing technology, and statistical method, to name a few.

With the reduction in sequencing costs, comparative transcriptomics has significantly broadened the potential for inter-species comparisons. The comparison of gene expression patterns between species can provide critical insights into the mechanisms driving phenotypic change [13–15]. For example, similarities in transcription patterns may indicate conservation or constraints in regulatory mechanisms [16–18]. On the other hand, divergence in transcription patterns can provide insights into the impacts genetic variation may have on expression levels within species [19]. Unfortunately, the comparison of transcriptional responses between species can be complicated by annotation limitations and the availability of fully sequenced genomes.

Firstly, there may be multiple gene predictions for a given organism based on multiple annotations of reference genomes. Inconsistencies between reference genomes can lead to differences and biases in gene annotations and mismatches in functional pathway identification. Secondly, observed differences between gene expression patterns from independent studies (i.e., experiments that were not conducted as part of the same study) may simply reflect differences in technical factors such as data processing, annotation, assembly parameters and others.

Ideally, comparisons between independent datasets require consistent pre-processing, analysis, and generation of outputs based on specific tools so that biological explanations of observed differences and similarities in gene expression patterns are teased out from technical artifacts. This methodological consistency applies to comparisons of not only species from different phylogenetic lineages but also experiments on tissues and cell types from the same organism. However, from a researchers’ perspective, it can be complicated and time-consuming to learn the commands of running each tool and that of the intermediate processes within a complete, consistent, and coherent data processing and analysis workflow.

We present here a novel pipeline, called CoRMAP (Comparative RNA-Seq Metadata Analysis Pipeline), which was developed for cross-species comparisons of transcriptomes. It implements a standardized workflow for the de novo meta-analysis of existing and novel raw RNA-Seq datasets. Since CoRMAP ensures that all raw datasets are processed in the same way, it circumvents several technical problems that emerge in cross-study or cross-species analyses. Notably, since all species undergo the same de novo assembly protocols, all datasets are subjected to the same technical biases. Further, no biases or mismatches are introduced simply because one species has more complete annotation relative to the other species.

In order to ensure that expression levels of evolutionarily related genes are used in the expression analysis we implemented OrthoMCL in our pipeline [20]. This orthology search creates orthologous gene groups and allows comparison of expression levels of these groups between species and experimental groups. Our approach is different from traditional comparative transcriptomics analyses, where genes are either directly compared based on gene identifiers and names, or indirectly compared at a higher-level via pathways and focus on the putative function of genes [21]. Still, CoRMAP can be linked to existing functional annotation tools (GO, KEGG, etc.) for gene groups [22].

To demonstrate the functionality and performance of our pipeline, we processed two mouse brain transcriptome datasets from memory formation studies using CoRMAP [23, 24]. These two studies not only used two different versions of the mouse genome but also used different study designs, processing protocols and statistical analyses. Nonetheless, we show that CoRMAP is capable of consolidating some of the findings from these studies and identifying gene expression patterns correlated with learning and memory. In order to compare the performance of our pipeline to other comparative transcriptomics pipelines, we also analyzed the two mouse datasets using a published functional mapping approach [25] and compared DEGs between the two pipelines.

## Implementation

### Workflow

**Fig. 1:**
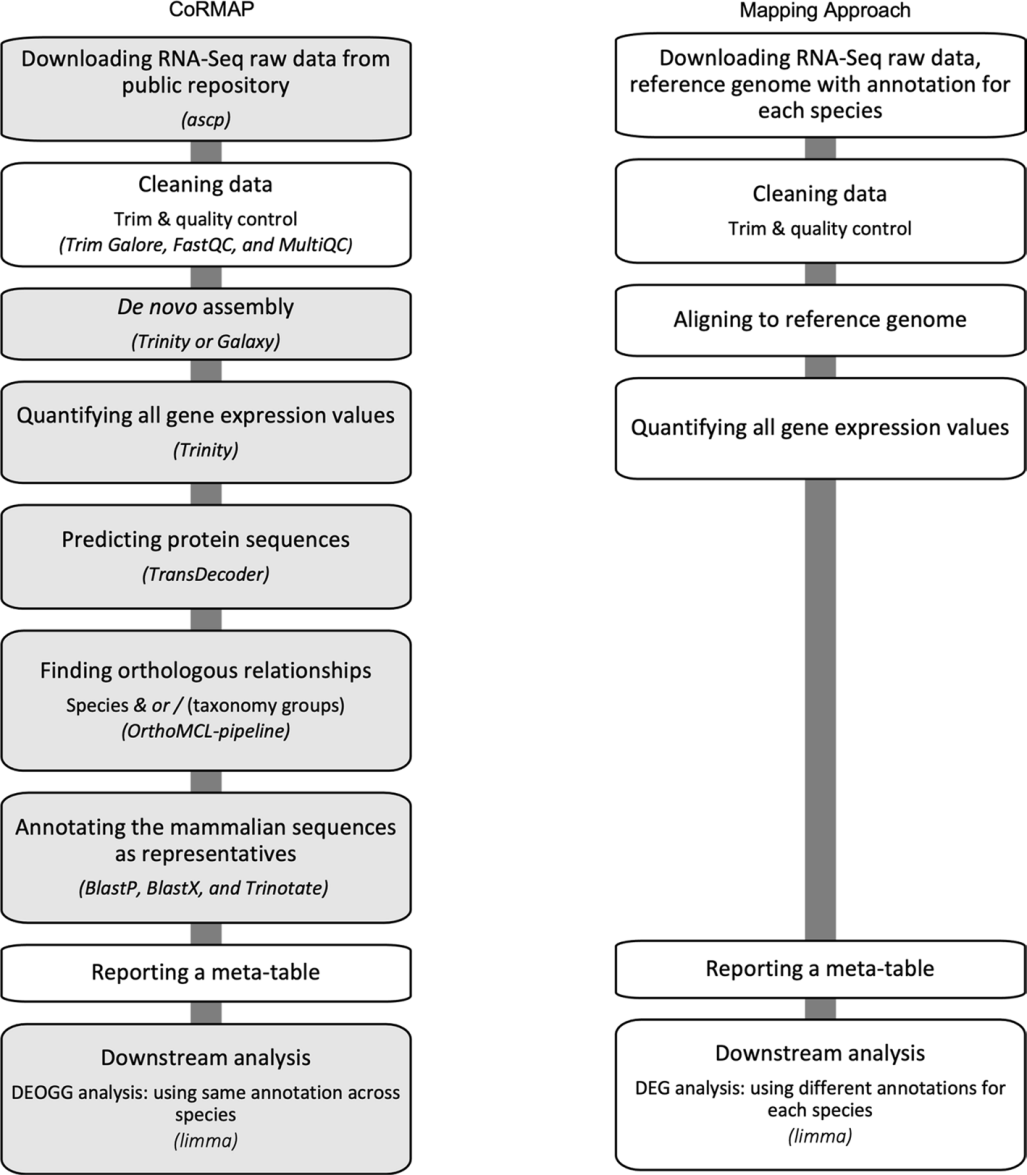
Flowcharts for CoRMAP (Comparative RNA-Seq Metadata Analysis Pipeline) and a mapping approach conducted in this study for comparison. The key software used in each step of CoRMAP is listed in italics in parenthesis after the description of steps

CoRMAP is implemented using several analysis tools for transcriptional data, and customized shell scripts and R scripts. This protocol provides a framework for conducting reference-independent comparative transcriptomic analysis across multiple species. CoRMAP obtains the assembled transcriptome for each species from raw transcriptome data, finds the orthologous relationships of coding genes across species or across a higher taxonomy group level, and plots expression patterns of orthologous gene groups across species. As such, there are three main data processing steps in CoRMAP: (1) de novo assembly, (2) ortholog searching, and (3) analysis of OGG expression patterns across species (or a higher taxonomy level). The complete workflow is illustrated in Fig. 1. Note that these analyses can be run together or separately, depending on the user’s needs. Because individual modules are combined within our pipeline, checkpoints for preliminary data analysis exist between each module. Below we provide a detailed summary of the individual steps within CoRMAP and instructions on how to implement the pipeline.

### Input

#### Installation of CoRMAP

For the installation of CoRMAP, execute the following command in a Linux terminal by downloading the repository from GitHub (git clone—https://github.com/rubysheng/CoRMAP.git). CoRMAP requires dependencies including ascp (https://www.ibm.com/docs/en/aci/3.9.2?topic=macos-ascp-transferring-from-command-line-ascp), FastQC (http://www.bioinformatics.babraham.ac.uk/projects/fastqc [26, 27]), MultiQC [26], Trim Galore! (v 0.6.4), Trinity (v2.8.6), TransDecoder (v 5.5.0) [27], Trinotate (v 3.2.1) (http://trinotate.github.io [28]), and OrthoMCL [28].

#### Preparation of datasets

CoRMAP includes a utility to download datasets of RNA-Seq raw data containing multiple runs from the Sequence Read Archive (SRA) [29, 30] database in batch by the software ascp. The RNA-Seq dataset of each project in the SRA database has a unique accession number that is recommended to be used as the directory name for each dataset when setting up the folder structure. Using the SRA accession numbers, FTP links are retrieved from the European Nucleotide Archive [29] and used as input to download the FASTQ files. As such, each FTP link refers to a compressed FASTQ file for a run. Every sample of the single-read sequencing method contains one run, while the sample of the paired-end sequencing method has two runs. As is standard practice, users are required to conduct quality checks on files used in this pipeline. This includes but is not limited to experimental design, replication and biological processing of samples.

#### Computational requirements and alternative options

The computing resources requirement for this pipeline is a large-memory server. Especially for the de novo assembly, this step requires about 1 Gb of RAM per 1 M reads to be assembled. Another option is to run some steps of this pipeline on Galaxy (http://usegalaxy.org), an open source and web-based bioinformatics platform.

For the orthologous searching, the normal hardware requirements include at least 4 Gb memory and about 100 Gb free space. There is an option to separate the calculation into multiple steps, depending on the number of datasets and the workstation hardware requirement. All parameters used in this pipeline can be found in the Additional file 1.

#### Trimming or quality control

RNA-Seq data quality control, including low-quality base calls trimming, adaptor auto-detecting and trimming, and short reads filtering, is performed by Trim Galore! using the default parameter settings. After this filtering, the minimum length of reads is 20 bp.

### Data processing

#### De novo assembly

The general processing of RNA-Seq raw data without reference genome consists of three steps: (i) normalization, (ii) gene assembly, and (iii) quantification and generation of the associated expression matrix. To reduce the number of input files, computational complexity and processing time, data normalization and de novo assembly by Trinity are separated into two steps. The targeted maximum coverage for reads is 50, while the k-mer size and the maximum percent of the mean for the standard deviation of k-mer coverage across reads are set at the default values of 25 and 200, respectively. Assemblies are assessed by basic contig N50 statistics, which specifies the length of the shortest contig that can cover 50% of the total genome length. The pipeline also has the options for users to compare assemblies with reference genomes and to assess the de novo assembly with QUAST [31].

#### Quantification

Transcript mapping and quantification back to the assembly are performed in Trinity using the plugin package RNA-seq by Expectation–Maximization (RSEM) [32, 33]. This plugin is used to estimate the abundance of contigs in an alignment-based algorithm with corrections for transcript lengths. In brief, RSEM assumes that each sequence read and read length are observed (one end for single reads and both ends for paired reads) and are generated according to a directed acyclic graph that includes parent sequence, length, start position and orientation. A Bayesian version of the expectation–maximization algorithm is used to obtain maximum likelihood estimates of the model parameters, transcript fractions and posterior mean estimates of abundances. The resulting gene expression matrices are then normalized to Transcripts Per Kilobase Million (TPM) to make them comparable across samples. The original TPM matrix generated from a Trinity plugin includes all gene names in each dataset. For each dataset, the expression matrix would be transformed into a long format with gene names, sample names and TPM values.

We also include the option to calculate TMM (Trimmed Mean of the M-values) values if desired. It is recommended to conduct standard descriptive statistics on TMM and/or TPM values, including but not limited to, outlier detection via PCA plots with replicates, expression levels analysis for extreme expression levels within replicates and the analysis of internal controls, if available. Subgroup structure of the data can also be revealed by the multidimensional scaling (MDS) plot using R package DESeq2 (v1.28.1) [34].

#### Orthologous gene searching

Genes that have an orthologous relationship are grouped using an automated pipeline for OrthoMCL [28, 35–37], called Orthomcl-pipeline (https://github.com/apetkau/orthomcl-pipeline), which requires the input of the amino acid sequences. Before running OrthoMCL for OGG identification, TransDecoder (Home TransDecoder/TransDecoder Wiki (github.com) is used to predict coding regions from assembled transcripts, thereby providing the amino acid sequences. For generating an efficient indexing system for the transcripts, we modified headers of transcripts by incorporating their original project accession numbers and their species codes. If users intend to compare expression data not only at the species level but also at a higher taxonomy level (e.g., families, genera, phyla, etc.) it is necessary to add the taxon code to the header of transcripts together with the species code.

An all-versus-all BLAST search with open reading frames is used to derive scores for pairwise sequence similarities. The E-value cut-off needs to be modified by the user and depends on the taxonomic distance between species or groups. To identify the ortholog or in-paralog pairs, each pair of matched sequences is filtered with 50% “percent match length” score. The percent match length score is obtained by counting the number of amino acids in the shorter sequence that participate in any High-scoring Segment Pair (HSP), dividing that by the length of the shorter sequence, and multiplying by 100. The filtered pairs are linked across or within the objects (species or class). To group orthologous genes, we implemented the Markov Clustering (MCL) algorithm which is included in the Orthomcl-pipeline. The output of the Orthomcl-pipeline includes a list of groups, including the orthologous protein sequence headers used to extract corresponding protein sequences, transcript sequences, quantification numbers and gene mapping information, for further analysis.

When searching for orthologs among several distantly related species, datasets can be split into groups based on their taxonomic relationships to reduce computational requirements. Then, orthologous gene searching (clustering) should be performed at two levels, intraclass- and interclass-. The general process is: (1) in-parallel orthologs searching within each class; (2) filtering clustered genes from those OGGs that contain orthologous genes from all species in one class; (3) integrating filtered genes from all classes for interclass orthologs searching; and (4) filtering clustered genes from those OGGs that contain orthologous genes from all classes.

If the input datasets require interclass orthologs searching followed by filtering, the output provides a cross-class OGG (cOGG). Due to the high annotation quality of the human and some other mammalian genomes, only clusters from these genomes are extracted for the annotation. Annotation of these Mammalia proteins (using BlastP) and transcripts (using BlastX) are obtained using Trinotate and the UniProt database as default. BlastP only returns the best match, while BlastX returns the top 5 hits. E-value cut-offs can be modified by the user depending on the evolutionary distance between organisms compared in the study. Other parameters in Trinotate and UniProt were set to defaults except for the parallel threads, which were set to use the maximum number of cores. Protein sequences are also searched against a protein profile HMM database and Trinotate is used to generate the final report.

#### Analyze expression patterns of orthologous gene groups

Based on the header name, CoRMAP extracts the expression values of orthologous genes from total transcripts in each dataset. Transcript variants refer to different versions of a transcript from the same gene in our pipeline. These, along with isoforms, are averaged within the same OGG group to represent the OGG expression value. For the final report, CoRMAP provides a meta-table with OGG number, gene name, annotation, and source information including species name, and a set of additional meta data such as tissue type (example e.g., brain region), study focus (example e.g., memory type) and study design. Once we obtain the meta-table, users can analyze and compare expression levels from different perspectives such as taxonomic groups, project design and tissue type, along with a variety of other experimental conditions. As mentioned above, the pipeline offers several checkpoints before calculating expression levels and before conducting comparisons between species.

## Results

CoRMAP processes RNA-Seq raw data to characterize gene expression patterns across species and higher taxonomic groups. Genes are clustered into orthologous gene groups and taxonomic groups are compared at the OGG level. CoRMAP also provides the option to incorporate other factors such as study design, taxonomic group, tissue type, etc., into the orthology search to facilitate taxonomic comparisons.

We compared two studies that were designed to analyze brain gene expression patterns during long-term learning and memory formation, specifically in the context of fear memories. Bero et al. [23] (PRJNA252803) studied the rapid response of the medial prefrontal cortex (mPFC) to memory encoding triggers, while Rao-Ruiz et al. [24] (PRJNA529794) studied the late-stage response of the dentate gyrus (DG) of the hippocampus genes to memory consolidation. Herein we refer to these studies as MT (for memory trigger) and MC (for memory consolidation) respectively. The analysis was performed using all default parameter settings, and with E-value cut-off of 10^–3^_._ Some immediate files were kept in the CoRMAP package as the template files.

Due to the different sequencing technologies used in these studies the total number of base pairs included in each assembly showed more than 20 times difference in coverage, ranging from 22.37 Mbp from MT to 461.68 Mbp from MC (Table 1). The minimum length of contig covering 50 percent of the assembled genome sequence for MT is about 2 times that of MC. As an example, we present a dataset in the Additional file 2, where we compared MT with reference genome mm39 (https://www.ncbi.nlm.nih.gov/assembly/GCF_000001635.27), having 0.566% of aligned bases in the reference genome with 1.053 duplication ratio. A duplication ratio over 1 indicates that the total number of aligned bases in the assembly was more than the total number of aligned bases in the reference genome. Both MT and MC are the short read only assembly, which has the feature of high sequence identity and high fragmentation unable to recreate the same structure of the genome.

**Table 1:** Summary results of CoRMAP applied to mouse brain datasets MT and MC

Statistic	MT	MC
Total base pairs (Mbp)	22.37	461.68
Contig N50 length (nt)	988	531
Average of transcripts per samples (K)	4.14	25.44
Total of transcripts (K)	33.08	966.56
Total number of predicted proteins (K)	12.57	120.77
Number orthologous protein sequences	9,556	18,225
Number unique orthologous genes	9,552	17,904
Number BlastX-annotated orthologous transcripts	9,024	17,354

To remove redundancy, CoRMAP decoded 38% of the total number of transcripts from MT to predicted protein sequences and 12% from MC. This common pool of sequences was used for orthologs searching based on the default E-value of 10^–3^. Although the input number of proteins from MC for orthologs searching was nearly 10 times larger than that of MT, the output of orthologous genes from MC was only 1.9 times larger than that of MT. From the total of 7407 OGGs we found between the two studies, 7047 unique OGGs were annotated by BlastX using the auto-generated unique protein SwissProt database, and the high quality reviewed and non-redundant protein sequence database in UniProt, from Trinotate (Table 1).

Downstream analysis of unique OGGs involves differential expression analysis between the two mouse studies. For each dataset, the expression value of each orthologous gene group was represented by averaging expression values of all genes within an OGG. The averaged expression values of the OGGs were then used to generate a new expression matrix at the OGG level. As both studies considered here had multiple treatments, the sample comparison pairs were classified either as Control (no learning) or Treatment (learned fear memory conditions). In order to gain insight into how many differentially expressed genes (DEGs) were retrieved from the original study, we manually matched the names of DEGs from original papers to our annotated OGGs.

The differential expression analysis of each dataset was performed using the same p-value threshold and adjustment method to their original study design and the results are summarized in Table 2. For MT and MC, we matched 51 and 455 OGGs, respectively. In MT, two OGGs (*FABP7* and *LMO7*) were identified as differentially expressed in our OGG expression matrix with the non-adjusted *p*-value < 0.05 cut-off. Using log fold-change with base 2, *FABP7* was down-regulated (Log_2_FC < − 1.00) while *LMO7* was up-regulated (Log_2_FC > 1). Two additional OGGs, *RPP29* and *CRIM1*, were identified at a 5% level of significance.

**Table 2:** DEOGGs identified by CoRMAP and found in the list of DEGs in the original MC paper. FC means the fold-change relative to Control using TPM (Transcripts per million) expression value as default. The reported p-value (at a 5% level) is FDR adjusted

Gene names	Log_2_FC	*p*-value
*MFGM*	− 3.36	0.03
*IBP2*	− 3.06	0.05
*CIR1*	− 2.43	0.02
*HAP1*	− 2.24	0.04
*ICLN*	− 2.06	0.04
*MPC2*	− 1.70	0.01
*PDCD6*	2.64	0.02
*SYNE1*	2.75	< 0.01
*GPR19*	3.27	0.01
*HSF2*	4.12	0.03
*ARC*	4.86	< 0.01

In MC, there were 11 differentially expressed OGGs (DEOGGs) from our OGG expression matrix identified by the differential expression analysis (FDR-adjusted p-value cut-off as 0.05), which were annotated with *ARC*, *SYNE1*, *GPR19*, *MPC2*, *PDCD6*, *CIR1*, *HSF2*, *MFGM*, *ICLN*, *HAP1*, and *IBP2*.

Finally, we performed the same differential expression analysis (no-adjusted *p*-value < 0.05 cut-off) in MT's and MC’s OGG matrices and found 24 common differentially expressed OGGs using a 5% *p*-value cut-off (Table 3). In the Additional file 3, we provide the results calculated by the expression values from TMM (Trimmed Mean of M-values). The TMM expression values were calculated using a between-sample normalization method while TPM expression values were calculated using within-sample normalization. Thus, MT and MC’s common DEOGGs detected by the TMM normalization method were distinct from those detected by the TPM normalization method.

**Table 3:** DEOGGs identified by CoRMAP in both MC and MT by the TPM and TMM (Trimmed Mean of M-values) expression value. FC means the fold-change relative to Control. The reported *p*-value (at a 5% level) is non-adjusted. OGGs that were not differential expressed filtered by p-value from TMM were represented with a dash

Gene	Group	MT				MC			
Name	Number	Log_2_FC(TPM)	*p*− value (TPM)	Log_2_FC (TMM)	*p*− value (TMM)	Log_2_FC(TPM)	*p*− value (TPM)	Log_2_FC (TMM)	*p*− value (TMM)
*T4S1*	10,016	− 1.10	0.02	− 1.10	0.02	− 2.01	0.04	− 7.55	0.00
*ESS2*	10,246	1.01	0.03	1.01	0.03	2.09	< 0.01	1.98	0.01
*WDR92*	12,036	− 1.59	0.01	− 1.59	0.01	3.59	< 0.01	1.77	0.02
*G137C*	12,213	0.82	0.02	0.82	0.02	2.11	0.03	− 3.67	0.02
*CAH2*	14,602	− 0.62	0.04	–	–	− 3.13	< 0.01	–	–
*ECHB*	2726	− 0.54	0.03	–	–	− 2.55	< 0.01	–	–
*RBM28*	3334	− 0.90	0.03	− 0.90	0.03	2.24	< 0.01	2.40	0.01
*STRN*	34,631	0.77	0.04	0.77	0.04	− 1.81	0.02	− 1.76	0.05
*FAS*	34,817	0.96	0.04	–	–	2.63	0.01	–	–
*LMO7*	34,823	1.11	0.04	1.11	0.04	1.87	0.01	7.05	0.00
*BD1L1*	34,841	1.38	0.01	1.40	0.01	− 2.96	< 0.01	− 5.47	0.00
*PCDH1*	34,903	0.86	0.04	0.86	0.04	2.62	0.01	− 3.61	0.02
*GCSP*	34,931	− 1.01	0.05	− 1.01	0.05	− 2.24	0.03	− 6.81	0.00
*MYCB2*	35,154	1.83	0.00	1.82	0.00	1.10	0.04	5.54	0.00
*DCNL4*	4130	0.55	0.04	–	–	− 1.68	0.01	–	–
*ST2B1*	4606	− 0.88	0.05	–	–	3.70	< 0.01	–	–
*CRIM1*	4717	0.69	0.02	0.69	0.02	2.92	< 0.01	− 2.90	0.01
*UNC80*	5200	0.98	0.01	0.98	0.01	2.58	< 0.01	1.49	0.03
*SLIRP*	5326	− 0.44	0.05	–	–	− 1.11	0.02	–	–
*FCRL2*	6903	1.01	< 0.01	–	–	3.19	0.00	–	–
*GPC5B*	6970	− 0.47	0.04	− 0.47	0.04	1.87	0.02	− 1.57	0.04
*MLC1*	8131	− 0.48	0.04	− 0.48	0.04	− 3.05	< 0.01	− 4.11	0.00
*ASTRB(ASTRA)*	8230	0.92	0.01	–	–	1.68	0.03	–	–
*TAU*	8242	0.49	0.04	0.49	0.04	− 1.36	0.01	1.84	0.03

In order to compare our method to alternative, mapping based approaches, we ran the two datasets through a functional mapping approach [25]. From this mapping approach, 179 common DEGs (Additional file 4) were found in MT and MC when data were normalized by TMM and filtered by no-adjusted *p*-value < 0.05 cut-off. We compared the list of common DEGs by the mapping approach to the 24 DEOGGs identified by CoRMAP and found one common gene – *LMO7*. 146 of the 178 remaining DEGs had nonmatched annotation from all OGGs’ annotation, while 15 DEGs matched to the OGGs that contained more than one gene for at least one mouse dataset and 17 DEGs matched to the OGGs that contained only one gene per dataset.

## Discussion

We introduced CoRMAP, a Linux-terminal-based pipeline designed for meta-analysis of comparative transcriptomics. CoRMAP consolidates the processing and analysis steps of RNA-Seq raw datasets from the SRA database into a pre-defined workflow. CoRMAP includes downloading raw data, structuring folders, improving the sequencing data quality, generating quality control reports, normalizing the transcripts to reduce duplicates (the same transcript sequence), de novo assembly, aligning and quantifying the gene expression, searching for orthologous gene groups, annotating, reporting a master table, and, finally, conducting differential expression analysis. In this paper, the downstream analysis produced the tables and charts to help interpret comparative transcriptomic analysis.

We applied the main procedure of this pipeline to two mouse studies that focused on fear induced memory formation to discover the shared orthologous gene expression patterns between the early stage of memory encoding and the late stage of memory consolidation. Based on previous work in this field, we assumed that the learning paradigm likely has the strongest impact on gene expression levels across studies [38, 39]. Thus, we re-analyzed two datasets using CoRMAP that were designed to identify differential gene expression in mouse brains in response to fear induced learning and compared the results of our analysis with the original published results.

The workflow of processing RNA-Seq raw data in this study is consistent with the widely used non-model organisms transcriptome profiling processes so that reference genomes would not be needed for either model or non-model animals [39–42]. Standardization and normalization were consistently applied to make datasets from different studies and different species comparable. Unlike traditional meta-analysis of transcriptome data, we reanalyzed the public transcriptome profiles related to learning and memory to enforce a standardized processing workflow starting from the RNA-Seq raw data. In addition, we tested the expression pattern of genes that were defined as DEGs from the original papers by CoRMAP with the same p-value cut-off and adjustment methods.

CoRMAP uses a novel annotation method whereby OGGs would be annotated only by rats or mice. This annotation standardization method is beneficial for some downstream analysis, such as GO annotation and enrichment, but requires at least one rat or mice dataset as the annotation representative. The consistency of annotated species could avoid missed or mistaken matches between input queries and mapping subjects. The occurrence of mismatch errors might be due to the inconsistent matching between function and gene identifier across species.

This clustering of OGGs is also helpful for simplifying the problem of variants and paralogs as we used the predicted protein sequences from transcripts to search the orthologous relationships. Although the clustering of OGGs is based on sequence similarity, the translated protein sequences representing the function of genes are used to inform the alignment. The initial goal of clustering OGGs was to cluster functionally equivalent genes. It is beneficial to group unknown genes with known genes from other species, resulting in genes from non-model organisms that can then be compared to well-annotated model species.

Compared to the alternative mapping approach in [21], our de novo approach recovered significantly fewer differentially expressed genes. This discrepancy can primarily be explained by the fact that the functional mapping pipeline used the latest mouse reference genome (mm39). Using a reference genome, if available, can increase the alignment completeness and accuracy, providing more mapped genes than de novo assembly used in our approach. However, until May 02, 2022, there have been only 119,373 species with a complete reference genome. Furthermore, functional mapping approaches that are applied to transcriptomes from non-model species without reference genomes can result in incorrect comparisons of genes (i.e. gene that are not orthologous), a problem our pipeline avoids by conducting orthogroup assignments [43]. Therefore, our pipeline provides an integrated tool that is compatible with functional mapping approaches and allows broad comparison of species while not requiring the presence of high-quality reference genomes.

By comparing TMM and TPM values, we found some substantial differences in the results. The TMM method is using a weighted trimmed mean of the log expression ratios across samples, effectively normalizing expression levels between conditions. In contrast, TPM values are primarily affected by sequencing depth and gene length showing the exact expression values.

Our analysis revealed three major findings: (1) there are substantial discrepancies between studies designed to analyze the same process using different designs and approaches. In fact, in the two datasets we used, no similarities in DEGs would be identified if the same statistical cut-offs were used. (2) CoRMAP analysis retrieves DEGs that can be compared to the DEGs of the original studies, so CoRMAP can be useful for the re-analysis and validation of existing datasets. (3) CoRMAP analysis was able to identify DEGs matching OGGs, indicating that our approach could be useful for the meta-analysis of transcriptional studies to identify candidate genes involved in biological processes. These target genes can then be used for functional validation in future studies.

In the DEG analyses, a *p*-value < 0.05 threshold was used for the MT data, while an FDR-adjusted *p*-value < 0.05 threshold was used for the MC data, to facilitate comparison of the CoRMAP identified DEGs with those identified in the original study analyses. However, the choice of p-value or FDR-adjusted *p*-value is somewhat controversial (Colquhoun, 2017). First, the false positive rate associated with a test can be much higher than the observed p-value; hence false positive rates should be reported along with p-values, or tests should be based on false positive rates. Second, the frequently used Benjamini–Hochberg FDR (1995) is flawed, as it is based on the number of rejected tests that would give rise to a p-value less than or equal to the observed p-value, the FDR only provides a minimum for the false positive rate. Instead, one could use Monte Carlo sampling to estimate the false positive rate as the proportion of rejected tests out of the set of tests with p-values *equalling* the observed p-value. While simulating average expression levels across OGGs to compute the Monte Carlo false positive rate is beyond the scope of this paper, caution should be exercised in the choice of p-value cut-off for DEG analysis of OGGs.

Hsieh et al. (2019) showed that algorithms developed for de novo transcriptome assembly, such as Trinity, rnaSPAdes [42, 43] and Trans-ABySS [44], have different implications for quantification depending on the assembly completeness and sequence ambiguity. In short, incomplete and over-extended contigs could lead to unreliable estimation of transcript abundance. The estimated abundance of a family-collapse contig often reflected the total expression of the collapsed transcripts and was close to the transcript generating the most amount of RNA reads. In contrast, for duplicated contigs, quantification error depended on how the algorithm allocated the RNA reads across the contigs. They recommended to use the total abundance of the contigs in a connected component in order to get accurate estimation. Analyses performed in CoRMAP downstream from assembly may inherit the biases introduced by the assembly algorithm. However, since all inputted RNA-Seq datasets are subjected to the same normalization, de novo assembly and quantification, any detected differences between control and treatment groups cannot be attributed to differences in method of normalization, assembly or quantification.

CoRMAP is an initial step towards creating a flexible pipeline for comparative transcriptome analysis. All components used are freely available and can be customized as needed. For example, popular assemblers such as SPAdes [44, 45] and Trans-ABySS [46] can be incorporated into the pipeline. With the integrated master table feature, users have the flexibility to perform any number of downstream analyses because CoRMAP appends the expression values with a diverse array of meta data from the studies used. Future analyses could take advantage of this framework for retrieving and comparing gene expression levels from diverse studies on a range of organisms and implement a principal component or clustering approach for the isolation of specific factors that contribute to discrepancies between studies.

## Conclusions

We developed and validated a versatile and powerful RNAseq processing pipeline for the comparative analysis of transcriptomes. This pipeline allows users to conduct de novo assemblies of published and novel datasets in order to mine for similarities and differences in expression patterns between species and higher taxonomic groups. The explicit comparative scope of the pipeline provides critical insights into similarities and divergences in gene expression patterns and can therefore help elucidate the evolutionary changes in gene expression and regulation across distantly related species.

## Supplementary Information

**Additional file 1**.** Supplementary Table 1**. A configuration table listing key parameters used in CoRMAP.

**Additional file 2**. Report.

**Additional file 3**. Trimmed Mean of M-values.

**Additional file 4**. Mapping approach, 179 common DEGs were found in MT and MC when data were normalized by TMM and filtered by no-adjusted *p*-value < 0.05 cut-off.

## Data Availability

RNA-seq raw data can be retrieved from the Sequence Read Archive (SRA) data (SRA Accession PRJNA252803 and PRJNA529794). https://github.com/rubysheng/CoRMAP.git. Project name: CoRMAP. Project home page: https://github.com/rubysheng/CoRMAP. Operating system(s): Ubuntu 16.04.7 LTS. Programming language: Bash and R. Other requirements: please go to https://github.com/rubysheng/CoRMAP/blob/mus_comparison/doc/Install.md. License: GNU GPL. Any restrictions to use by non-academics: licence needed.

## Abbreviations

cOGG: Cross class orthologous gene group
CoRMAP: Comparative RNA-Seq metadata analysis pipeline
DEG: Differentially expressed gene
DEOGG: Differentially expressed orthologous gene group
DG: Dentate gyrus
GO: Gene ontology
HSP: High-scoring segment pair
KEGG: Kyoto encyclopedia of genes and genomes
Mbp: Mega base pairs
MC: Memory consolidation
MCL: Markov clustering algorithm
MDS: Multidimensional scaling
mPFC: Medial prefrontal cortex
MT: Memory trigger
nt: Nucleotide
OGG: Orthologous gene group
RNA-Seq: High throughput RNA sequencing technologies
RSEM: RNA-seq by expectation–maximization
SRA: Sequence read archive
TMM: Trimmed mean of the M-values
TPM: Transcripts Per Kilobase million
