# Supplementary material for: Comparative transcriptomics analysis pipeline for the meta-analysis of phylogenetically divergent datasets (CoRMAP)
Source: BMC Bioinformatics. 2022 Oct 7;23:415. doi: 10.1186/s12859-022-04972-9 (PMC9547434; doi:10.1186/s12859-022-04972-9)
Supplement: Supplementary file 2 — Additional file 2. Report. [file 12859_2022_4972_MOESM2_ESM.pdf]

Report

	Trinity
# contigs (>= 0 bp)	33081
# contigs (>= 1000 bp)	5882
# contigs (>= 5000 bp)	97
# contigs (>= 10000 bp)	1
# contigs (>= 25000 bp)	0
# contigs (>= 50000 bp)	0
Total length (>= 0 bp)	22374227
Total length (>= 1000 bp)	11097703
Total length (>= 5000 bp)	590903
Total length (>= 10000 bp)	11310
Total length (>= 25000 bp)	0
Total length (>= 50000 bp)	0
# contigs	13305
Largest contig	11310
Total length	16279237
Reference length	2728222451
GC (%)	46.56
Reference GC (%)	41.67
N50	1448
N75	868
L50	3403
L75	7078
# misassemblies	17269
# misassembled contigs	5871
Misassembled contigs length	8378633
# local misassemblies	11896
# scaffold gap ext. mis.	0
# scaffold gap loc. mis.	0
# unaligned mis. contigs	83
# unaligned contigs	13 + 335 part
Unaligned length	456883
Genome fraction (%)	0.566
Duplication ratio	1.053
# N's per 100 kbp	0.00
# mismatches per 100 kbp	142.58
# indels per 100 kbp	19.67
# genomic features	260729 + 245209 part
Largest alignment	10159
Total aligned length	15067421
NA50	788
NGA50	-
NA75	329
LA50	5728
LA75	13138

All statistics are based on contigs of size >= 500 bp, unless otherwise noted (e.g., "# contigs (>= 0 bp)" and "Total length (>= 0 bp)" include all contigs).

Misassemblies report

	Trinity
# misassemblies	17269
# contig misassemblies	17269
# c. relocations	16662
# c. translocations	603
# c. inversions	4
# scaffold misassemblies	0
# s. relocations	0
# s. translocations	0
# s. inversions	0
# misassembled contigs	5871
Misassembled contigs length	8378633
# local misassemblies	11896
# scaffold gap ext. mis.	0
# scaffold gap loc. mis.	0
# unaligned mis. contigs	83
# mismatches	21426
# indels	2956
# indels (<= 5 bp)	2182
# indels (> 5 bp)	774
Indels length	43494

All statistics are based on contigs of size >= 500 bp, unless otherwise noted (e.g., "# contigs (>= 0 bp)" and "Total length (>= 0 bp)" include all contigs).

Unaligned report

	Trinity
# fully unaligned contigs	13
Fully unaligned length	11558
# partially unaligned contigs	335
Partially unaligned length	445325
# N's	0

All statistics are based on contigs of size >= 500 bp, unless otherwise noted (e.g., "# contigs (>= 0 bp)" and "Total length (>= 0 bp)" include all contigs).

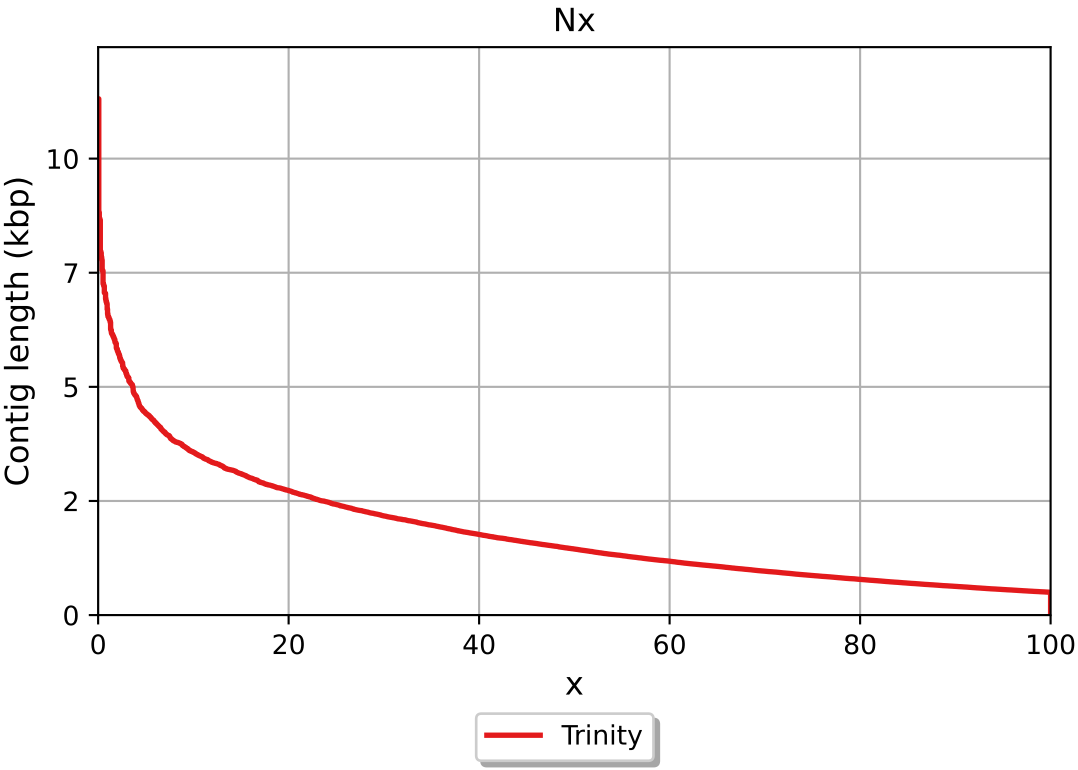

Nx
Contig length (kbp)
0
2
5
7
10
0
20
40
60
80
100
x
Trinity

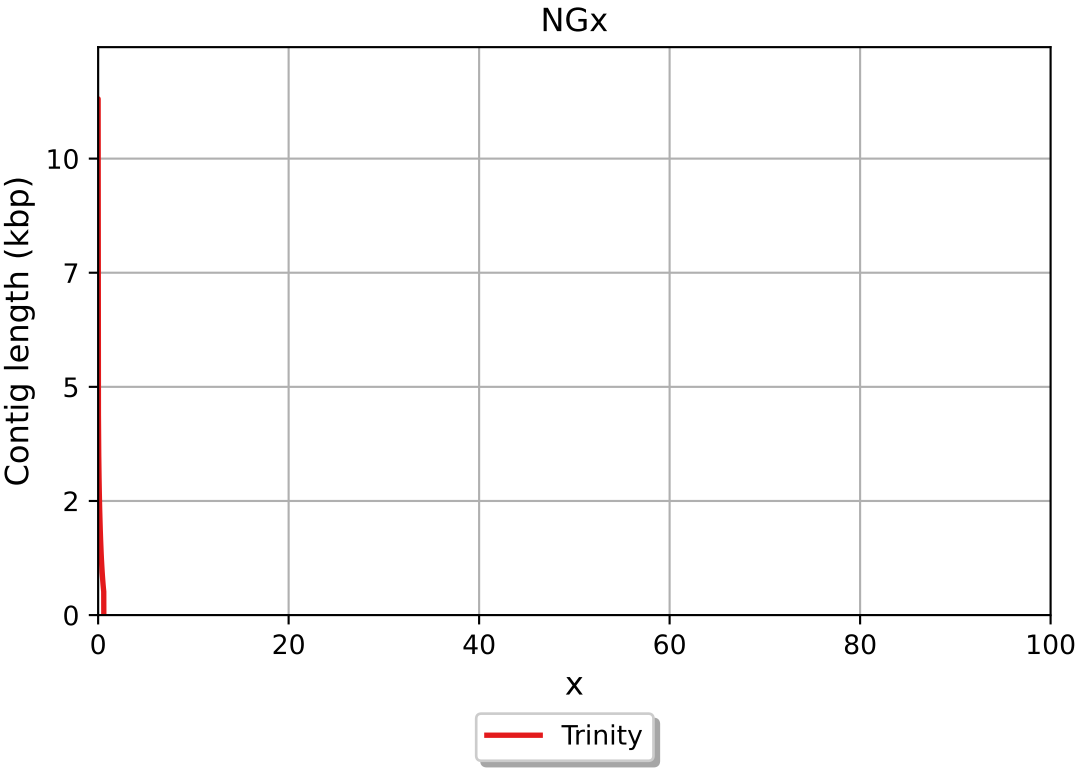
NGx
Contig length (kbp)
10
7
5
2
0
0
20
40
60
80
100
x
Trinity

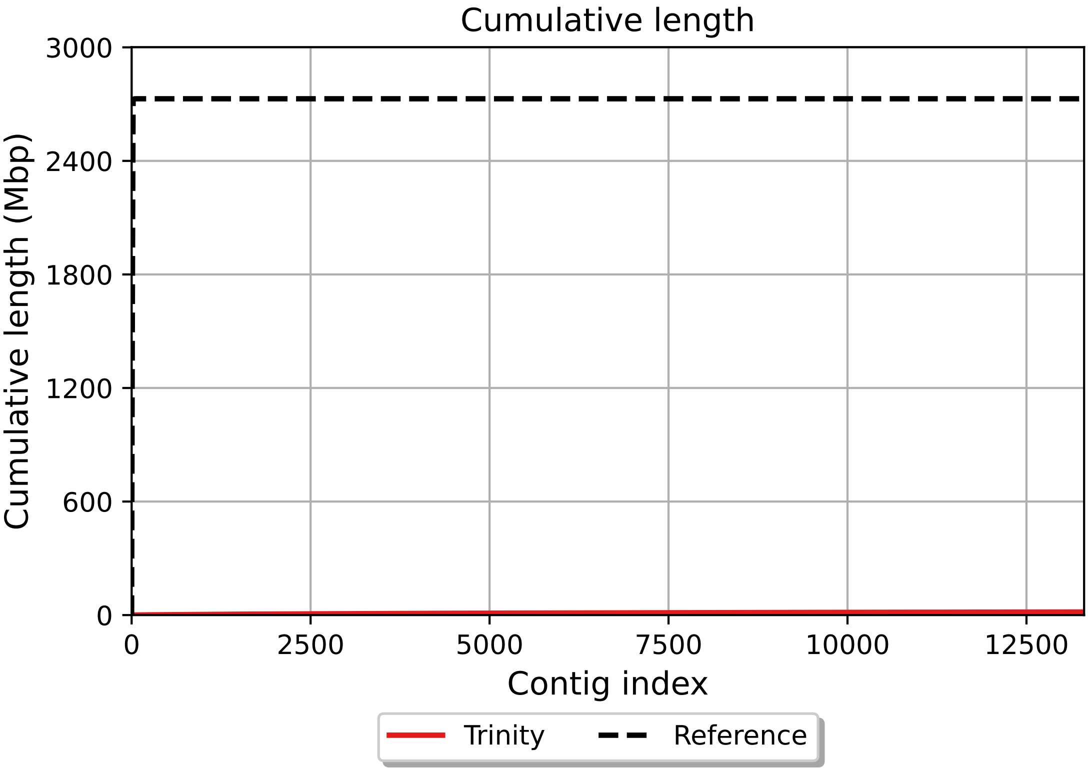

Cumulative length
Cumulative length (Mbp)
3000
2400
1800
1200
600
0
0
2500
5000
7500
10000
12500
Contig index
Trinity
Reference

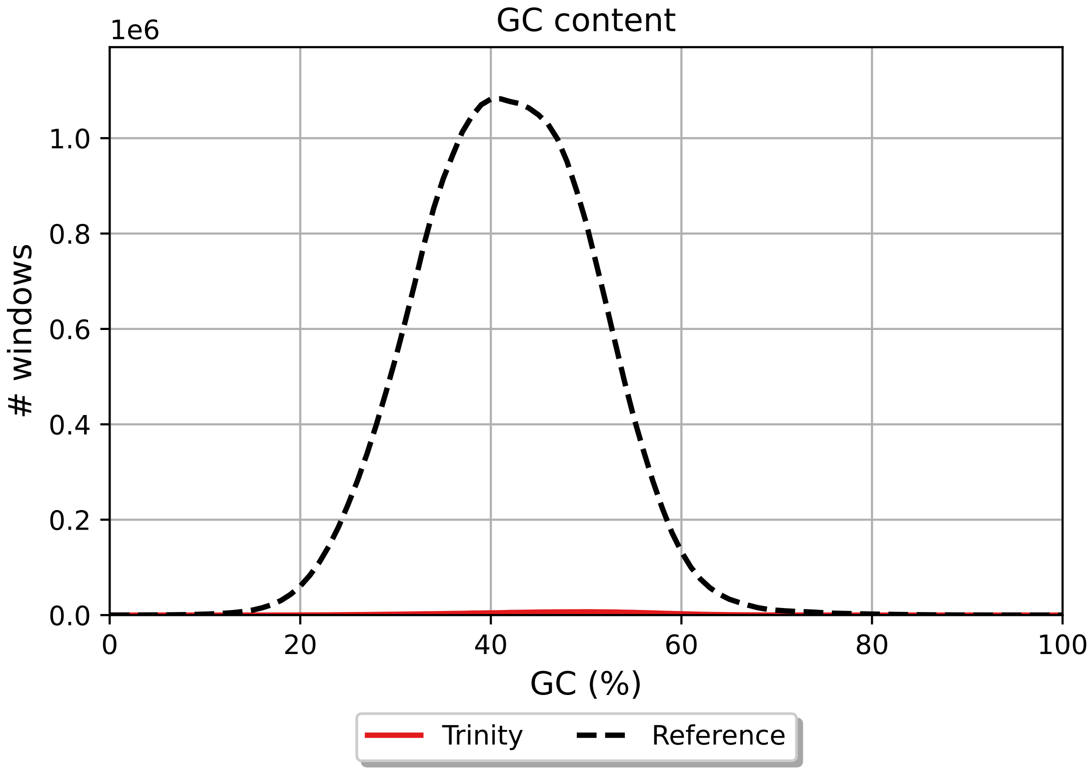
GC content
1e6
1.0
0.8
0.6
0.4
0.2
0.0
windows
0
20
40
60
80
100
GC (%)
Trinity
Reference

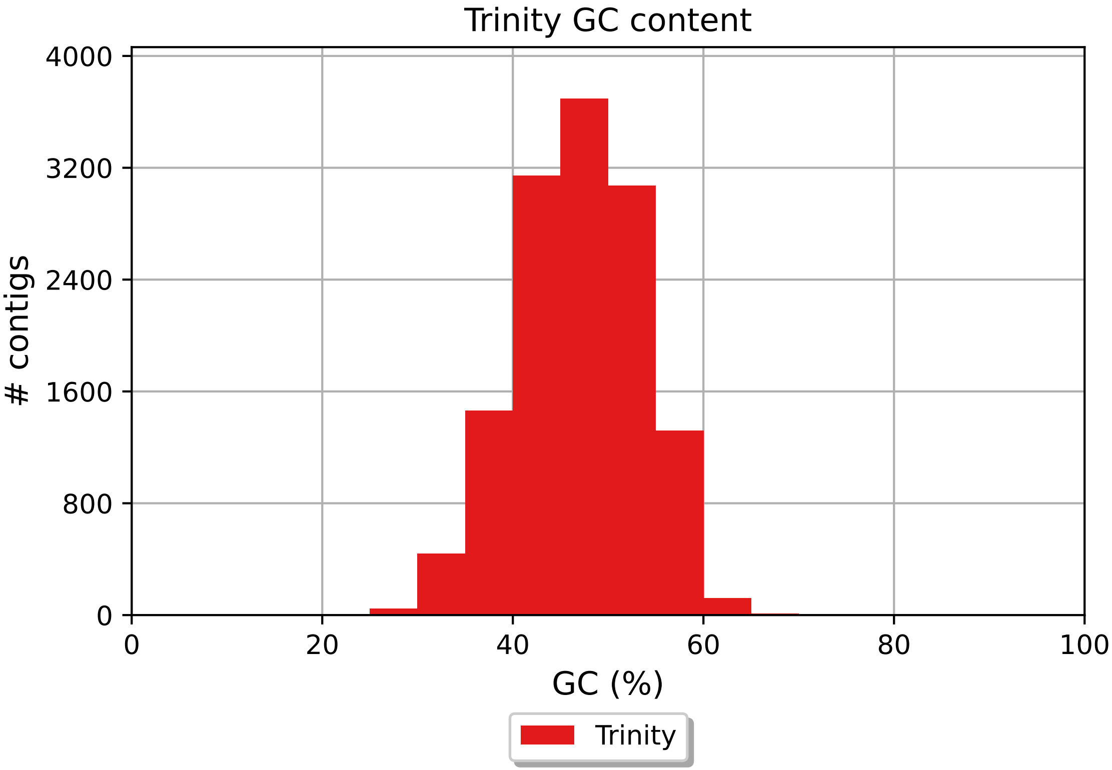
Trinity GC content
contigs
4000
3200
2400
1600
800
0
0
20
40
60
80
100
GC (%)
Trinity

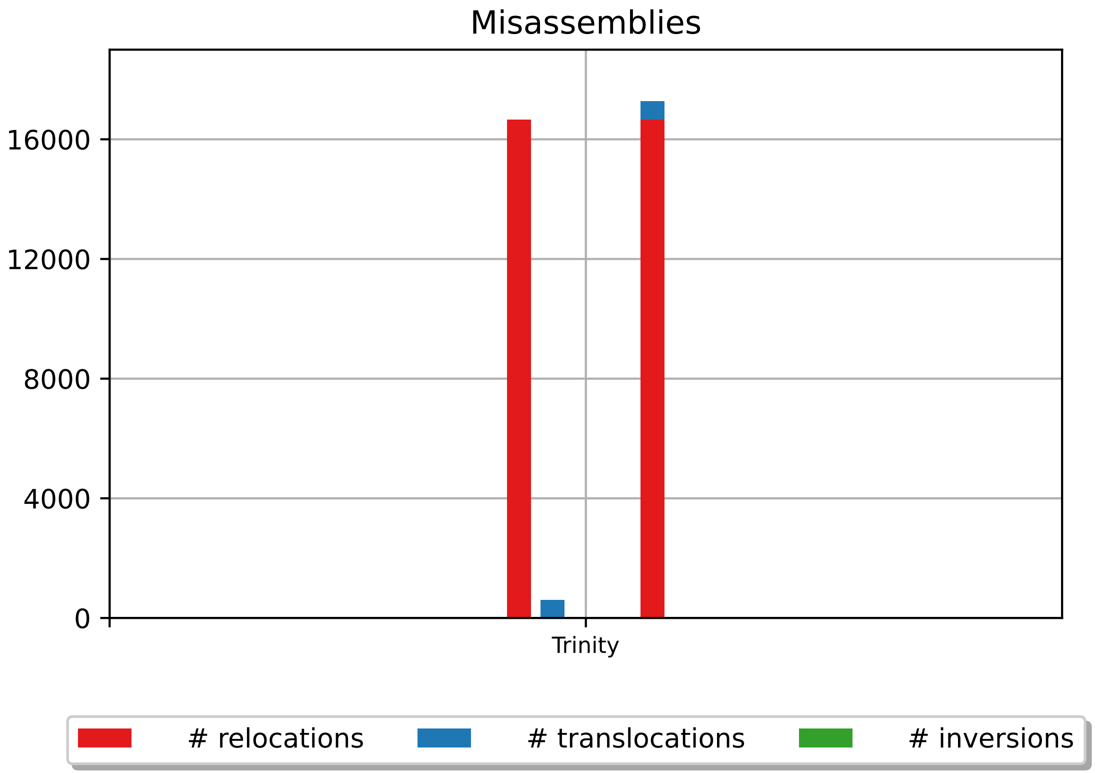
Misassemblies
16000
12000
8000
4000
0
Trinity
relocations
translocations
inversions

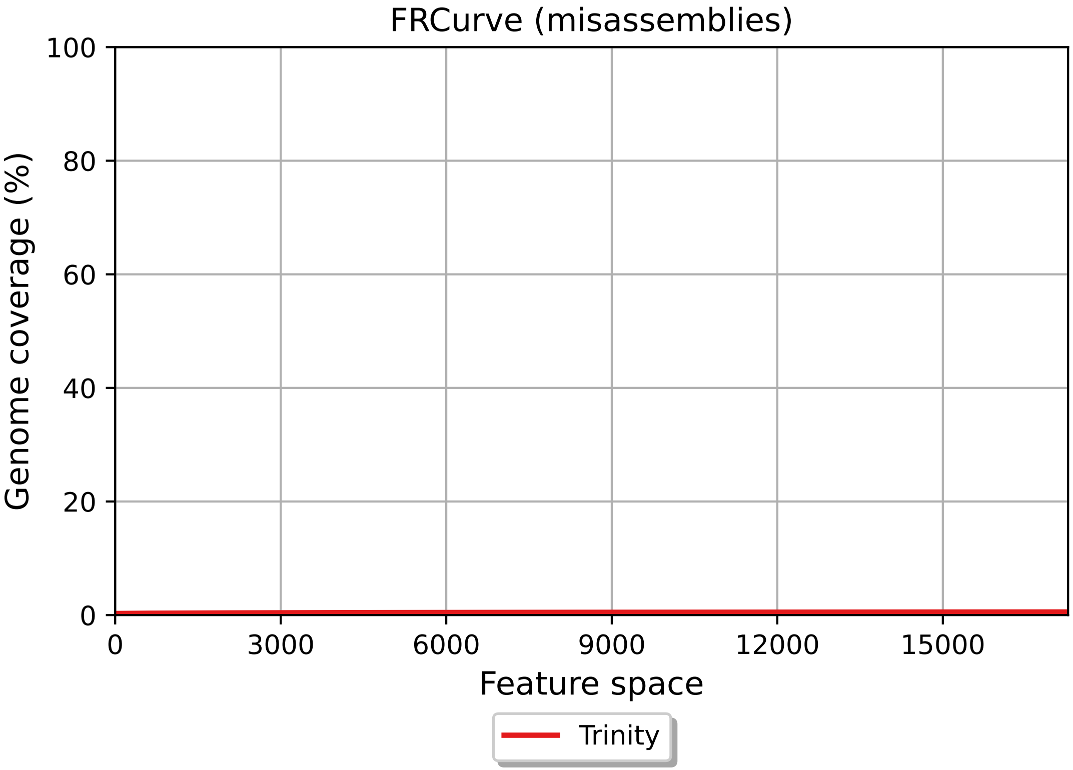
FRCurve (misassemblies)
Genome coverage (%)
100
80
60
40
20
0
0
3000
6000
9000
12000
15000
Feature space
Trinity

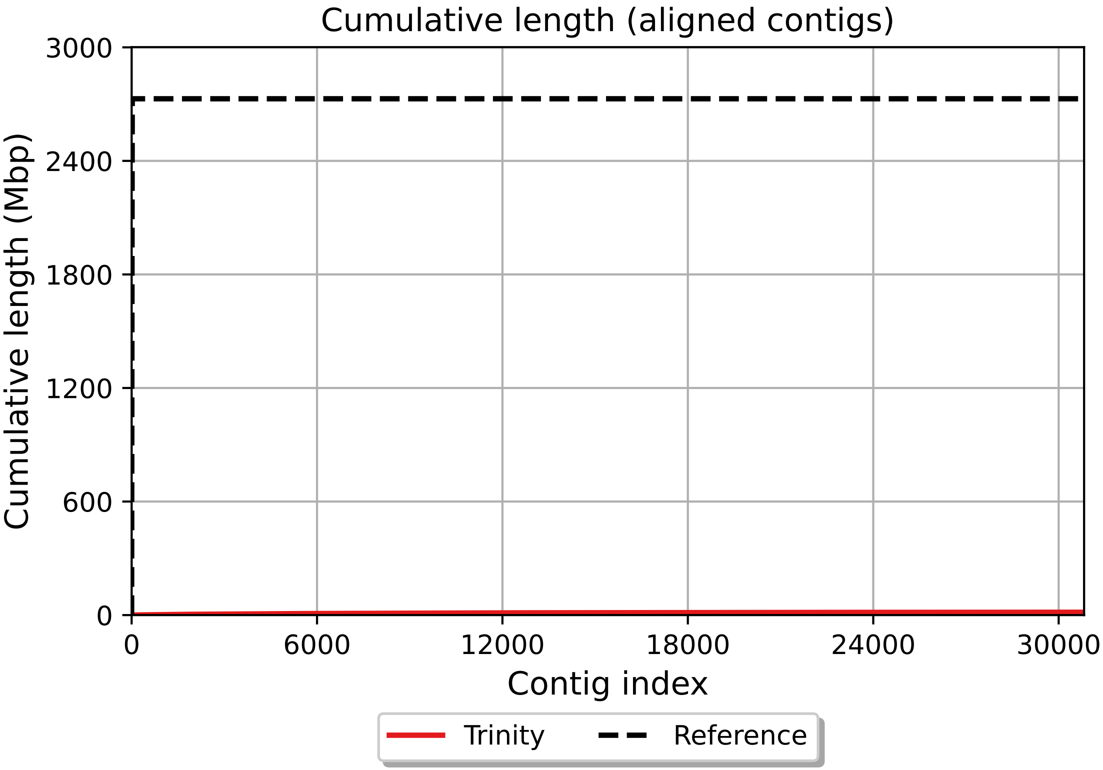
Cumulative length (aligned contigs)
Cumulative length (Mbp)
3000
2400
1800
1200
600
0
0
6000
12000
18000
24000
30000
Contig index
Trinity
Reference

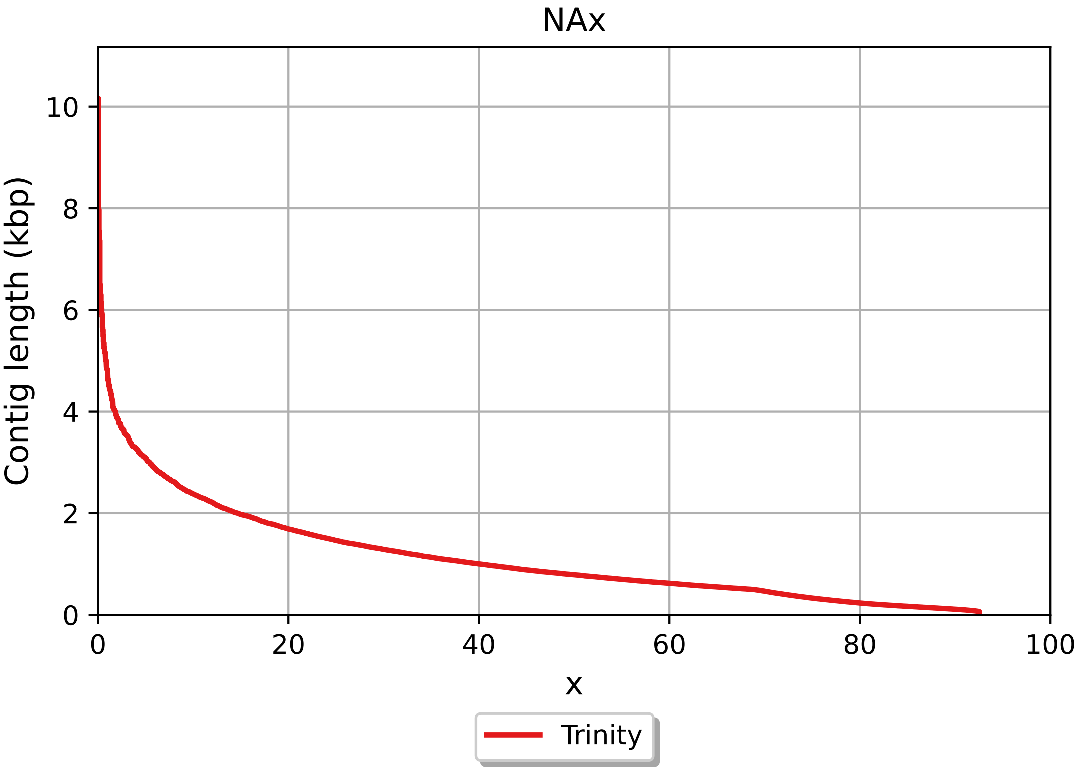

NAx
Contig length (kbp)
0
2
4
6
8
10
0
20
40
60
80
100
x
Trinity

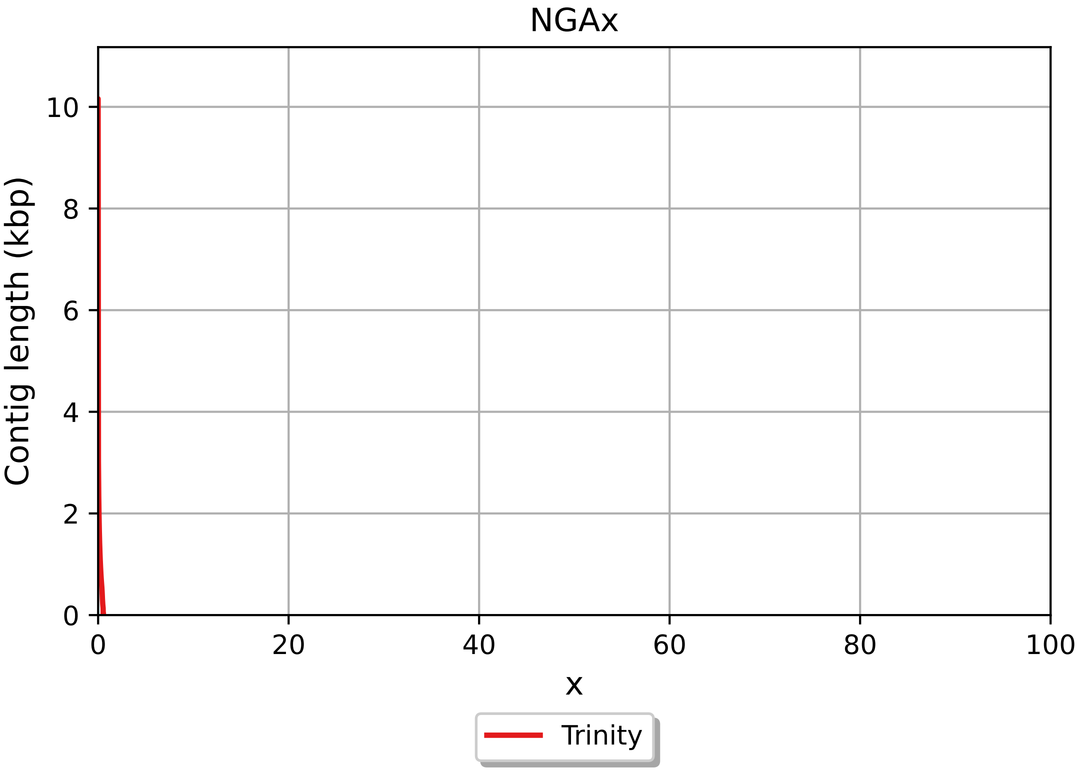
NGAx
Contig length (kbp)
0
2
4
6
8
10
0
20
40
60
80
100
x
Trinity

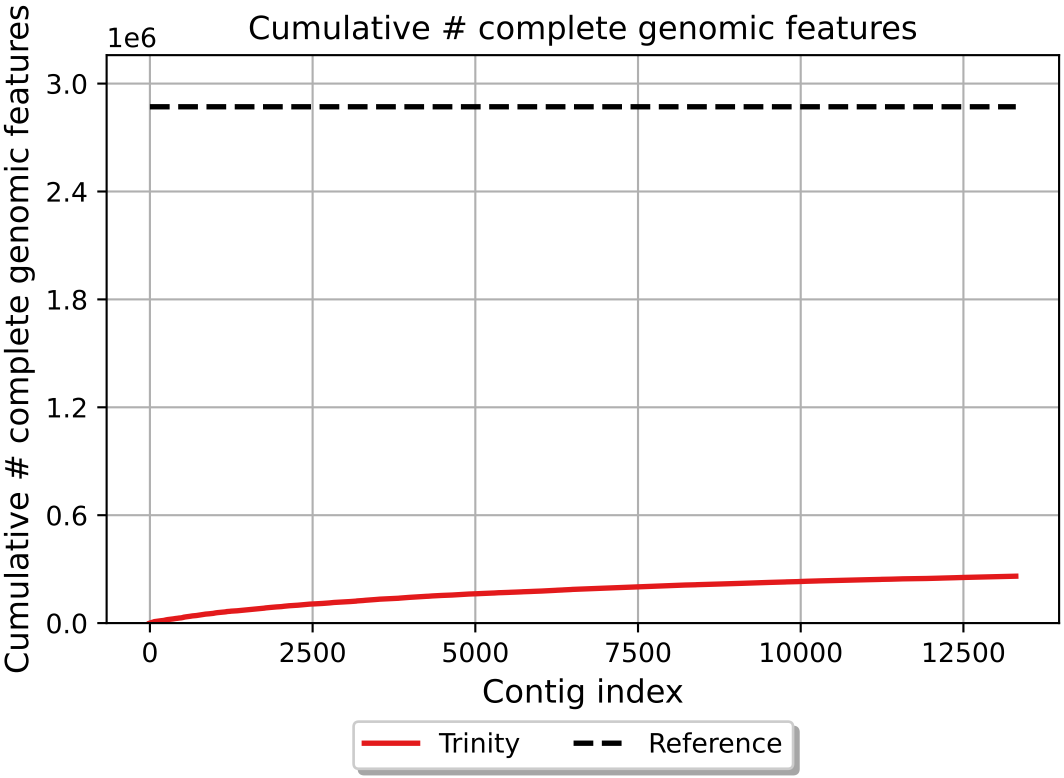

Cumulative # complete genomic features
1e6
3.0
2.4
1.8
1.2
0.6
0.0
Cumulative # complete genomic features
0
2500
5000
7500
10000
12500
Contig index
Trinity
Reference

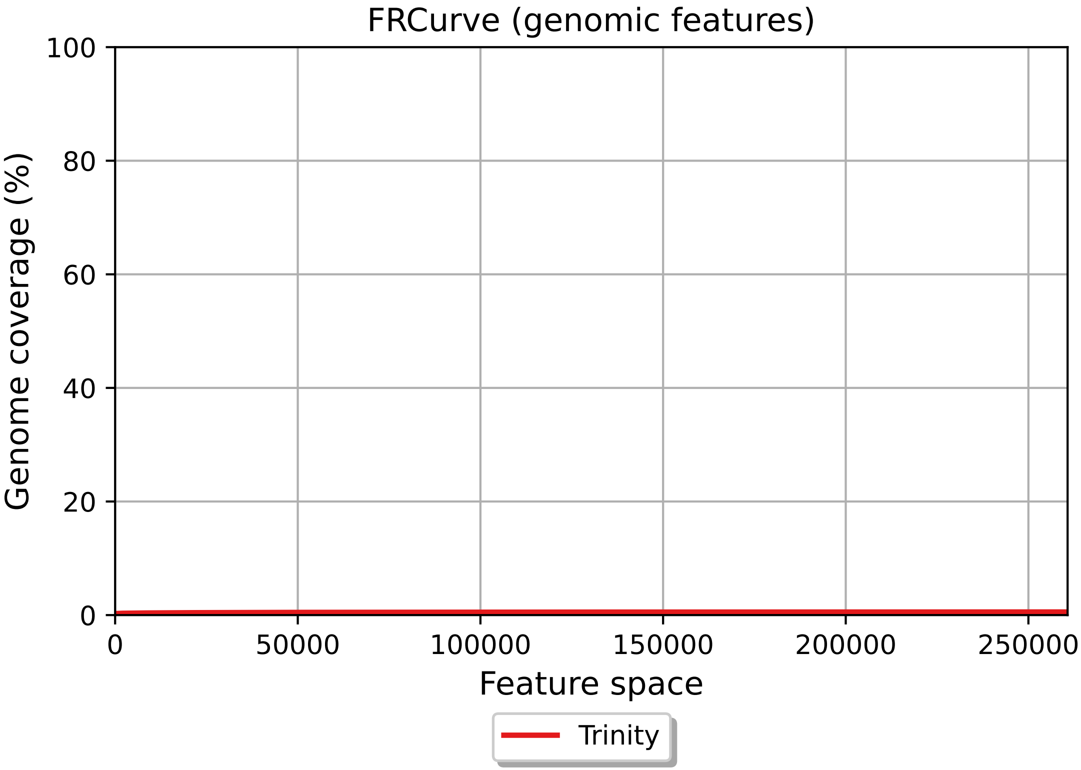
FRCurve (genomic features)
100
80
60
40
20
0
Genome coverage (%)
0
50000
100000
150000
200000
250000
Feature space
Trinity
